# Heart Dosimetric Parameters Were Associated With Cardiac Events and Overall Survival for Patients With Locally Advanced Esophageal Cancer Receiving Definitive Radiotherapy

**DOI:** 10.3389/fonc.2020.00153

**Published:** 2020-03-12

**Authors:** Guoxin Cai, Chuanbao Li, Jinming Yu, Xue Meng

**Affiliations:** ^1^Department of Radiation Oncology, Cheeloo College of Medicine, Shandong University, Jinan, China; ^2^Department of Radiation Oncology, Shandong Cancer Hospital and Institute, Shandong First Medical University and Shandong Academy of Medical Sciences, Jinan, China; ^3^Department of Emergency, Chest Pain Center, Qilu Hospital of Shandong University, Jinan, China

**Keywords:** heart dosimetric parameters, cardiac events, esophageal cancer, overall survival, definitive radiotherapy

## Abstract

**Objectives:** The aim of this study was to assess the association between heart dosimetric parameters and cardiac events or overall survival (OS) for patients with stage III esophageal cancer receiving definitive radiotherapy.

**Materials and Methods:** Patients with stage III esophageal cancer receiving definitive radiotherapy at our hospital from 2011 to 2013 were enrolled retrospectively. The primary endpoint was grade ≥ 2 cardiac events, and the second endpoint was 5-year OS. Competing risk analysis and Cox regressions analysis were performed to evaluate the association between heart dose and cardiac events or OS.

**Results:** Three hundred forty-six patients were analyzed. Median follow-up was 30 months. Median prescribed dose was 60 Gy. Seventy-eight patients (22.5%) had 91 grade ≥ 2 cardiac events, at a median of 14 months to first event. Thirty-three patients (9.5%) had 42 grade ≥ 3 cardiac events. Of the 78 patients with grade ≥ 2 cardiac events, 70 (89.7%) had the first cardiac events that occurred within first 3 years after radiotherapy. Multivariable analysis showed that preexisting ischemic heart disease [hazard ratio (HR), 2.26; 95% confidence interval (CI), 1.26–4.06; *p* = 0.006] and mean heart dose (HR, 1.12; 95% CI, 1.04–1.20; *p* = 0.002) were significantly associated with increased risk of grade ≥ 2 cardiac events. Disease progression (HR, 2.60; 95% CI, 1.82–3.70; *p* < 0.001), Eastern Cooperative Oncology Group (ECOG) performance status (HR, 0.71; 95% CI, 0.56–0.91; *p* = 0.007), heart volume receiving ≥ 5 Gy (V5, HR, 1.01; 95% CI, 1.00–1.03; *p* = 0.035), and gross tumor volume (GTV; HR, 1.00; 95% CI, 1.00–1.00; *p* = 0.020) were significant predictors of 5-year OS on multivariable analysis.

**Conclusion:** Higher heart dose was significantly associated with an increased cardiac event rate and a worse OS outcome for patients with stage III esophageal cancer treated with definitive radiotherapy. Most of the first cardiac events occurred within first 3 years after treatment.

## Introduction

With a long-term follow-up, increases in probabilities of cardiac toxicities with ionizing radiation exposure to the heart have been observed in long-term survivors of lymphoma (1, 2) and breast cancer (3, 4). For lung cancer receiving high-dose radiotherapy, studies have confirmed that clinically relevant cardiac complications are significantly associated with heart dose and occur fairly early after treatment (5, 6). The Radiation Therapy Oncology Group (RTOG) 0617 trial also confirmed that reduction of the heart dose was associated with improved overall survival (OS) (7). Concurrent chemoradiotherapy with or without surgery has become a standard treatment for patients with locally advanced esophageal cancer (8, 9). Cardiac doses are generally higher in esophageal cancer than breast cancer or Hodgkin's lymphoma owing to the location of the target area close to the heart and/or to the high total dose (10). However, given the typically long latency of radiation-associated cardiac toxicities, the relatively low incidence, and poor prognosis of esophageal cancer, studies are limited on radiation-induced cardiac toxicities for esophageal cancer. In fact, the incidence of cardiac events in esophageal cancer receiving radiotherapy is relatively high, and it may occur earlier than historically understood. Beukema et al. (10) summarized six reports on radiation-induced cardiac toxicity in esophageal cancer and found that the overall crude incidence of cardiac events was 10.8% (range: 5–44%) and most events occurred within 2 years after treatment. For patients with esophageal cancer, the addition of radiation therapy could increase the risk of cardiac death (11). However, studies regarding whether heart dosimetric parameters could independently predict radiation-associated cardiac toxicities for patients with esophageal cancer are very limited. Given it is a clinically observable and a common late complication, there is a pressing demand for seeking the most sensitive dose–volume parameters significantly associated with radiation-associated cardiac toxicities and further guiding the delineation of organs at risk in clinical practice.

In this study, we aimed to evaluate the incidence of cardiac events within 5 years after treatment and the relationship between heart dose–volume parameters and cardiac events or OS in stage III esophageal cancer patients treated with definitive radiotherapy.

## Materials and Methods

### Patients

**Figure 1:**
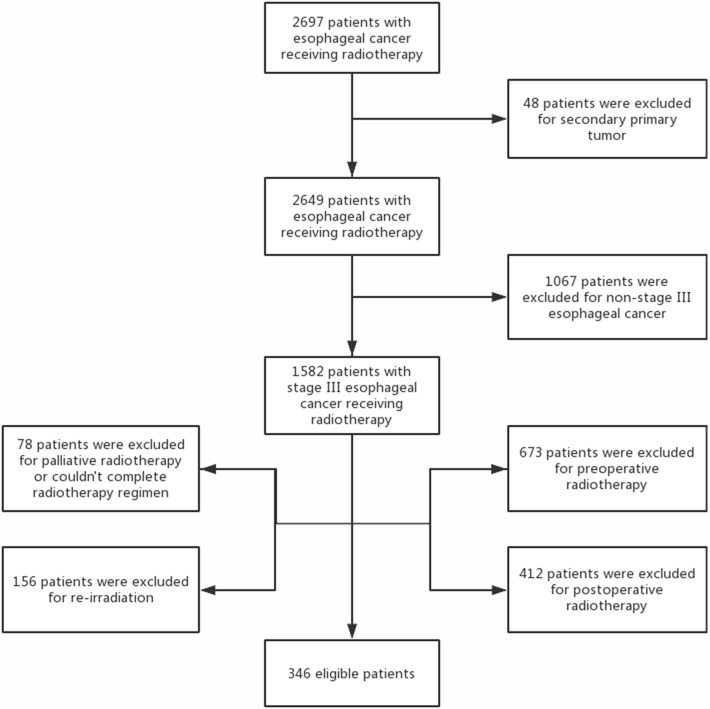
A flow diagram of patient selection.

A retrospective analysis was carried out for patients with stage III esophageal cancer and Eastern Cooperative Oncology Group (ECOG) performance status of 0–2 treated with definitive radiotherapy at Shandong Cancer Hospital and Institute between 2011 and 2013. Pretreatment evaluation included electrocardiogram (ECG), esophageal endoscopy with biopsy, endoscopic ultrasound, contrast-enhanced chest and abdomen computed tomography (CT), and bone scan. Tumor was staged according to the American Joint Committee of Cancer (AJCC) seven edition criteria (12). Those who received palliative, preoperative, and postoperative radiotherapy or re-irradiation to the thorax; could not complete radiotherapy regimen; or had secondary primary tumor were excluded from this analysis. A flow diagram of patient selection is shown in Figure 1. This study was approved by the Ethics Committee of Shandong Cancer Hospital and Institute, and informed consent was obtained from all included individuals.

### Treatment

The details of treatment plan were collected from medical records at our hospital. All patients received definitive radiotherapy, and most patients received two 3-weekly concurrent cycles of cisplatin and fluorouracil. Cisplatin (80 mg/m^2^) was infused intravenously on day 1, and 5-fluorouracil (1 g/m^2^ per day) was administered as a continuous infusion on days 1–4. Treatment-planning CT scans using intravenous contrast were performed for all patients. Gross tumor volume (GTV), defined as any visible primary tumor and metastatic regional nodes, was determined by radiation oncologists using all possible resources [barium esophagography, CT, positron emission tomography–CT (PET-CT), endoscopic ultrasound, etc.]. Clinical target volume (CTV) was defined as the GTV plus 3–5 cm superior and distal margin depending on tumor location and 3–5 mm radial margin. Supraclavicular lymph nodes were included electively for upper esophageal cancer, and celiac lymph nodes were included for distal esophageal cancer on the basis of the decision of radiation oncologists. The planning target volume (PTV) was defined as CTV plus a 1-cm margin in all directions. The radiation therapy was delivered at 1.8–2.2 Gy per fraction daily and five fractions per week by using linear accelerators with 6- or 10-MV energy of X-ray. The prescribed dose ranged from 50 to 66 Gy. Radiation techniques included three-dimensional conformal radiation therapy (3D-CRT) or intensity-modulated radiation therapy (IMRT). The normal tissue tolerance dose limits were set as follows: heart volume receiving ≥30 Gy (V30) ≤ 40%, mean heart dose ≤ 30 Gy, lung volume receiving ≥ 5 Gy (V5) ≤ 65%, lung volume receiving ≥ 20 Gy (V20) ≤ 30%, mean lung dose ≤ 20 Gy, and maximum dose to spinal cord ≤ 45 Gy.

### Toxicity Assessment and Follow-Up Endpoints

The primary endpoint was grade ≥ 2 cardiac events. The cardiac events were defined by certified cardiologists, and we obtained the details of cardiac events by inpatient and outpatient medical records. Cardiac events were graded according to the Common Terminology Criteria for Adverse Events (CTCAE) version 4.03. Malignant pericardial effusions were not regarded as cardiac events. Baseline cardiac risk was assessed by noting whether patients had preexisting ischemic heart disease, which was defined as the diagnosis of coronary artery disease (including acute myocardial infarction, angina pectoris, and ischemic heart failure) with or without coronary artery bypass grafting procedure, angioplasty, or stent placement. For patients without preexisting ischemic heart disease (*n* = 306), the World Health Organization/International Society of Hypertension (WHO/ISH) risk score (13) was calculated to assess patients' baseline cardiac risk. Current or former smokers were defined as those who smoked more than one cigarette per day and smoked for more than 5 years. Alcoholics were defined as those who consumed more than 25 g (15 g for woman) of alcohol per day and drank for more than 5 years. The second endpoint was 5-year OS. The last follow-up time was December 2018. Follow-up evaluation was performed every 3 months for the first 2 years after treatment and every 6 months thereafter by contrast-enhanced chest and abdomen CT scan, endoscopy, and barium esophagography.

### Statistical Analysis

Progression-free survival (PFS) and OS were estimated using the Kaplan–Meier method. The Fine and Gray competing risk regression model (14) was used to evaluate the cumulative incidence of cardiac events adjusted for the significant competing risk of death and to analyze the association between covariates and cardiac events on univariable and multivariable analyses. Consistent with the RTOG 0617 (7), we chose heart V5, heart V30, and heart volume receiving ≥ 60 Gy (V60) to represent low, medium, and high dose exposure, respectively. In this study, cervical and upper thoracic esophageal cancers were defined as upper tumor; midthoracic and lower thoracic esophageal cancers were defined as lower tumor. The univariable Cox regression analysis was used to analyze the association of covariates with 5-year OS. Dosimetric parameters including lung V5, lung V20, mean lung dose, heart V5, heart V30, heart V60, mean heart dose, and GTV were included in the univariate OS analysis. Clinical and dosimetric variables with a *p*-value < 0.10 on the univariable analysis were incorporated into the multivariable Cox regression analysis. *p*-value ≤ 0.05 was considered to be a significant difference. The Kaplan–Meier analysis and Cox regression analysis were performed with SPSS Statistics V23.0 (IBM Corporation, Armonk, NY, USA); Fine and Gray competing risk regression model was performed with R (version 3.6.0).

## Results

Patient characteristics are listed in Table 1. Median follow-up was 30 months. Median prescribed dose was 60 Gy. Most patients (85.5%) received concurrent chemotherapy. Forty patients (11.6%) had preexisting ischemic heart disease. For those without preexisting cardiac disease (*n* = 306), 149 (48.7%) had a WHO/ISH 10-year risk score of ≥20%.

**Table 1:** Patient characteristics.

**Characteristic**	**Number (%)**
**Patient characteristics**	
Gender	
Male	249 (72.0)
Female	97 (28.0)
COPD history	
Yes	35 (10.1)
No	311 (89.9)
Current or former smoker	
Yes	177 (51.6)
No	169 (48.4)
Alcoholic	
Yes	150 (43.4)
No	196 (56.6)
Diabetes mellitus	
Yes	26 (7.5)
No	320 (92.5)
Preexisting ischemic heart disease	
Yes	40 (11.6)
No	306 (88.4)
^*^WHO/ISH 10-year risk, %	
<20	157 (51.3)
≥20	149 (48.7)
ECOG performance status	
0	147 (42.5)
1	169 (48.8)
2	30 (8.7)
Histology	
Squamous cell carcinoma	336 (97.1)
Adenocarcinoma	3 (0.9)
Small cell carcinoma	7 (2.0)
T stage	
T1	9 (2.6)
T2	68 (19.6)
T3	249 (72.0)
T4	20 (5.8)
N stage	
N0	4 (1.2)
N1	163 (47.1)
N2	156 (45.1)
N3	23 (6.6)
Tumor location	
Cervical	22 (6.4)
Upper thoracic	135 (39.0)
Midthoracic	136 (39.3)
Lower thoracic	53 (15.3)
Median age at esophageal cancer diagnosis, year (SD)	66 (8.34)
**Treatment characteristics**	**Median (SD)**
Treatment modality, number (%)	
Concurrent chemotherapy	296 (85.5)
No chemotherapy	50 (14.5)
Radiotherapy technique, number (%)	
3D-CRT	110 (31.8)
IMRT	236 (68.2)
Median prescribed radiation dose, Gy	60 (4.10)
Median lung V5, %	56.81 (19.80)
Median lung V20, %	22.38 (6.93)
Median mean lung dose, Gy	12.44 (3.54)
Median max lung dose, Gy	65.71 (6.89)
Median lung volume, cm^3^	3,250.30 (1,033.44)
Median heart V5, %	44.12 (39.50)
Median heart V30, %	16.04 (24.34)
Median mean heart dose, Gy	12.26 (13.48)
Median heart volume, cm^3^	576.10 (169.45)
Median GTV volume, cm^3^	97.75 (112.77)

*COPD, chronic obstructive pulmonary disease; WHO/ISH, World Health Organization/International Society of Hypertension; ECOG, Eastern Cooperative Oncology Group; 3D-CRT, three-dimensional conformal radiation therapy; IMRT, intensity-modulated radiation therapy; V5, volume received ≥ 5 Gy; V20, volume received ≥ 20 Gy; V30, volume received ≥ 30 Gy; GTV, gross tumor volume; SD, standard deviation*.

* *The WHO/ISH 10-year risk was assessed only for patients without preexisting ischemic heart disease (n = 306)*.

### Cardiac Events

**Figure 2:**
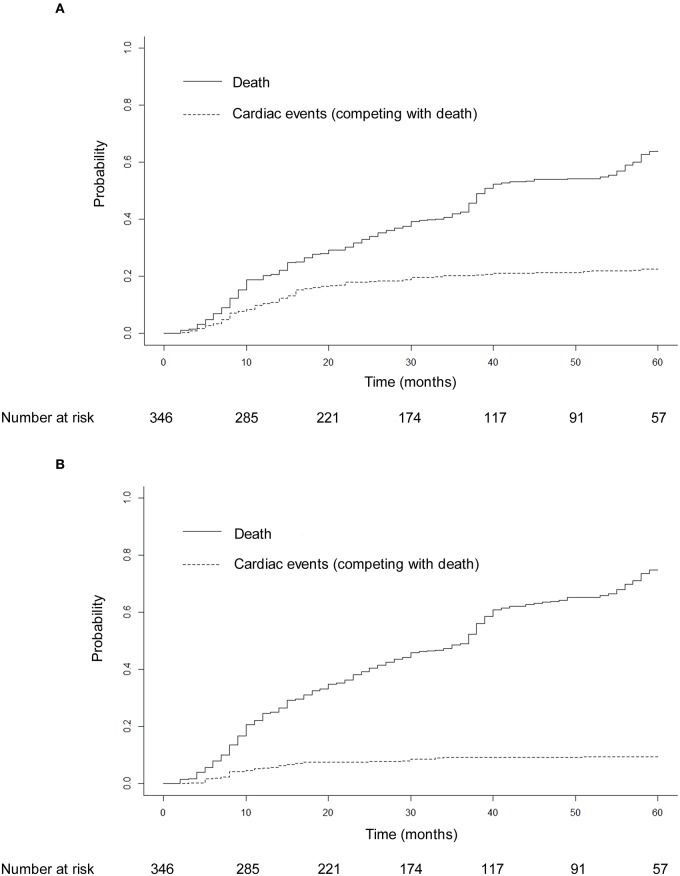
Cumulative incidence of grade ≥ 2 cardiac events **(A)** and grade ≥ 3 cardiac events **(B)** adjusted for the competing risk of death. After adjustment for the competing risk of death, the 3- and 5-year probabilities were 20.23 and 22.54% for grade ≥ 2 cardiac events and 9.25 and 9.54% for grade ≥ 3 cardiac events.

Seventy-eight patients (22.5%) had 91 grade ≥ 2 cardiac events at a median of 14 months to first cardiac events. Thirty-three patients (9.5%) had 40 grade ≥ 3 cardiac events. Three patients had grade 4 cardiac events: one pericardial tamponade case received urgent pericardiocentesis 34 months after treatment, one acute myocardial infarction case developed hemodynamic instability and received emergent percutaneous coronary intervention 16 months after treatment, and one ventricular fibrillation case received urgent electric defibrillation 2 months after diagnosis of acute myocardial infarction and 16 months after treatment. Five patients had fatal grade 5 cardiac events: two patients died of acute myocardial infarction 5 and 7 months after treatment, two patients died of heart failure 21 and 30 months after treatment, and one patient had fatal ventricular fibrillation 1 month after diagnosis of acute myocardial infarction and 18 months after treatment. One patient had four cardiac events, two patients had three cardiac events, and six patients had two cardiac events during follow-up. The details of cardiac events are displayed in Table 2 and Supplementary Table 1. The median dosimetric parameters of the patients who experienced grade ≥ 2 or grade ≥ 3 cardiac events are shown in Table 3. Of the 78 patients with grade ≥ 2 cardiac events, 70 (89.7%) had the first cardiac events that occurred within the first 3 years after treatment. Accounting for death as a competing risk, the 3- and 5-year probabilities were 20.23 and 22.54% for grade ≥ 2 cardiac events and 9.25 and 9.54% for grade ≥ 3 cardiac events. Figure 2 shows the cumulative incidence of grade ≥ 2 and grade ≥ 3 cardiac events.

**Table 2:** Cardiac event as graded by CTCAE version 4.03.

**Cardiac event**	**Grade 2**	**Grade 3**	**Grade 4**	**Grade 5**	**Grade ≥ 3**	**Total (%)**
Acute coronary syndrome	1	15	1	2	18	19 (20.9)
Arrhythmia	14	6	1	1	8	22 (24.2)
Heart failure	3	3	0	2	5	8 (8.8)
Pericardial effusion	23	2	0	0	2	25 (27.4)
Pericarditis	1	2	0	0	2	3 (3.3)
Pericardial tamponade	0	0	1	0	1	1 (1.1)
Valvular disease	9	3	0	0	3	12 (13.2)
Myocarditis	0	1	0	0	1	1 (1.1)
Total (%)	51 (56.0)	32 (35.2)	3 (3.3)	5 (5.5)	40 (44.0)	91 (100.0)

*CTCAE, Common Toxicity Criteria Adverse Events*.

**Table 3:** The median dosimetric parameters of the patients who experienced grade ≥ 2 or grade ≥ 3 cardiac events.

	**Median**		***p*-value**	**Median**		***p*-value**
	**Grade ≥ 2 cardiac events**	**Non-grade ≥ 2 cardiac events**		**Grade ≥ 3 cardiac events**	**Non-grade ≥ 3 cardiac events**	
Lung V5, %	58.73	58.61	0.326	66.81	56.55	0.120
Lung V20, %	22.38	22.39	0.848	24.33	22.33	0.775
Mean lung dose, Gy	12.50	12.41	0.661	14.63	12.40	0.283
Heart V5, %	68.54	40.68	<0.001	97.70	39.48	0.005
Heart V30, %	31.69	13.59	<0.001	44.08	13.23	<0.001
Heart V60, %	0.06	0.04	0.082	1.40	0.02	0.096
Mean heart dose, Gy	19.94	10.49	<0.001	27.16	10.39	<0.001

*V5, volume received ≥ 5 Gy; V20, volume received ≥ 20 Gy; V30, volume received ≥ 30 Gy; V60, volume received ≥ 60 Gy*.

The multivariable competing risk-adjusted analysis for grade ≥ 2 and grade ≥ 3 cardiac events is listed in Table 4. On the multivariable analysis, preexisting ischemic heart disease [hazard ratio (HR), 2.26; 95% confidence interval (CI), 1.26–4.06; *p* = 0.006] and mean heart dose (HR, 1.12; 95% CI, 1.04–1.20; *p* = 0.002) were significant predictors of grade ≥ 2 cardiac events for all patients. For those without preexisting ischemic heart disease (*n* = 306), mean heart dose (HR, 1.10; 95% CI, 1.0 2–1.19; *p* = 0.014) and WHO/ISH 10-year risk score (HR, 1.74; 95% CI, 1.01–3.00; *p* = 0.047) were significantly associated with increased hazard of grade ≥ 2 cardiac events. In the subgroup analysis, for patients with lower tumor (*n* = 192), mean heart dose was a significant predictor of grade ≥ 2 cardiac events whether for all patients or for patients without preexisting ischemic heart disease (Table 5). But for patients with upper tumor (*n* = 154), all of the dosimetric factors had no significant association with grade ≥ 2 cardiac events (data not shown).

**Table 4:** Multivariable competing risk-adjusted analysis for grade ≥ 2 and grade ≥ 3 cardiac events.

**Characteristic**	**Multivariable analysis (grade** **≥** **2 cardiac events)**				**Multivariable analysis (grade** **≥** **3 cardiac events)**			
	**All patients (*****n*** **=** **346)**		**Patients without preexisting ischemic heart disease (*****n*** **=** **306)**		**All patients (*****n*** **=** **346)**		**Patients without preexisting ischemic heart disease (*****n*** **=** **306)**	
	**HR** **(95% CI)**	***p*-value**	**HR** **(95% CI)**	***p*-value**	**HR** **(95% CI)**	***p*-value**	**HR** **(95% CI)**	***p*-value**
Preexisting ischemic heart disease	2.26 (1.26–4.06)	0.006			3.29 (1.42–7.61)	0.006		
^*^WHO/ISH 10-year risk ≥ 20, %			1.74 (1.01–3.00)	0.047			4.94 (1.23–19.89)	.025
Heart V5, %	0.99 (0.97–1.01)	0.311	0.99 (0.97–1.01)	0.285	1.04 (1.01–1.07)	0.011	0.98 (0.96–1.01)	0.158
Heart V30, %	0.97 (0.95–1.01)	0.101	0.98 (0.95–1.01)	0.216	1.02 (0.98–1.07)	0.253	0.97 (0.93–1.01)	0.125
Heart V60, %	0.97 (0.91–1.03)	0.289	0.97 (0.91–1.04)	0.388	1.03 (0.92–1.15)	0.587	0.95 (0.89–1.03)	0.212
Mean heart dose, Gy	1.12 (1.04–1.20)	0.002	1.10 (1.02–1.19)	0.014	0.92 (0.81–1.03)	0.153	1.17 (1.07–1.28)	0.001

*WHO/ISH, World Health Organization/International Society of Hypertension; V5, volume received ≥ 5 Gy; V30, volume received ≥ 30 Gy; V60, volume received ≥ 60 Gy; HR, hazard ratio; CI, confidence interval*.

* *The WHO/ISH 10-year risk was assessed only for patients without preexisting ischemic heart disease (n = 306)*.

**Table 5:** Multivariable competing risk-adjusted analysis for grade ≥ 2 cardiac events in patients with lower esophageal cancer.

**Characteristic**	**Multivariable analysis**			
	**All patients (*****n*** **=** **346)**		**Patients without preexisting ischemic heart disease (*****n*** **=** **306)**	
	**HR** **(95% CI)**	***p*-value**	**HR** **(95% CI)**	***p*-value**
Preexisting ischemic heart disease	3.16 (1.61–6.20)	0.001		
^*^WHO/ISH 10-year risk ≥ 20, %			1.99 (1.04–3.80)	0.037
Heart V5, %	0.99 (0.97–1.01)	0.173	0.98 (0.96–1.00)	0.095
Heart V30, %	0.98 (0.95–1.01)	0.147	1.00 (0.95–1.04)	0.849
Heart V60, %			0.95 (0.87–1.04)	0.269
Mean heart dose, Gy	1.10 (1.03–1.19)	0.006	1.11 (1.05–1.17)	<0.001

*WHO/ISH, World Health Organization/International Society of Hypertension; V5, volume received ≥ 5 Gy; V30, volume received ≥ 30 Gy; V60, volume received ≥ 60 Gy; HR, hazard ratio; CI, confidence interval*.

* *The WHO/ISH 10-year risk was assessed only for patients without preexisting ischemic heart disease (n = 306)*.

**Figure 3:**
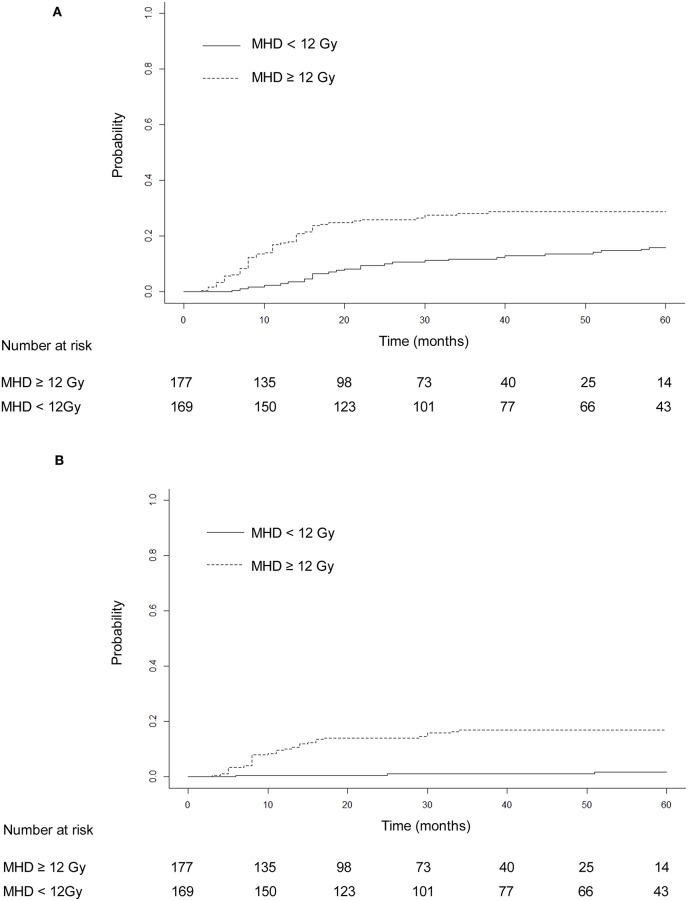
Cumulative incidence of competing risk-adjusted grade ≥ 2 cardiac events **(A)** and grade ≥ 3 cardiac events **(B)** in patients with heart mean dose ≥12 and <12 Gy. After adjustment for the competing risk of death, the 3- and 5-year rates of grade ≥ 2 events were, respectively, 28.25 and 28.81% for patients with mean heart dose ≥ 12 Gy and 11.83 and 15.98% for patients with mean heart dose <12 Gy (*p* = 0.045). The 3- and 5-year competing risk-adjusted rates of grade ≥ 3 cardiac events were 16.95 and 16.95% in patients with mean heart dose ≥ 12 Gy and 1.18 and 1.78% in patients with mean heart dose <12 Gy (*p* = 0.031).

**Figure 4:**
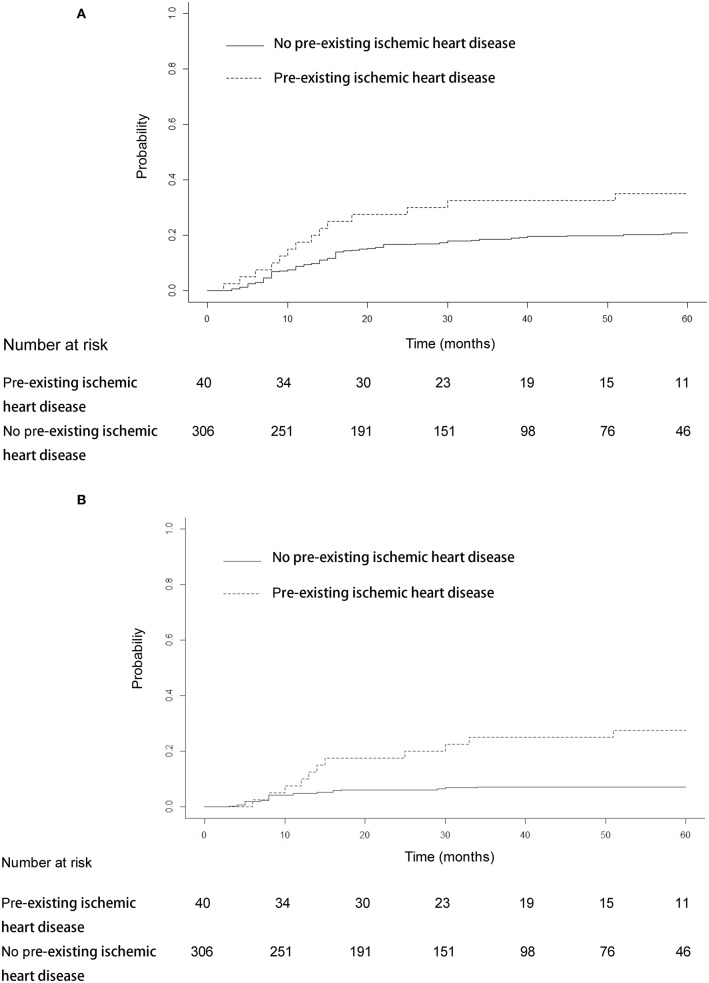
Cumulative incidence of competing risk-adjusted grade ≥ 2 cardiac events **(A)** and grade ≥ 3 cardiac events **(B)** in patients with and without preexisting ischemic heart disease. After adjustment for the competing risk of death, the 3- and 5-year probabilities of grade ≥ 2 cardiac events were 32.5 and 35% compared with 18.6 and 20.9% in patients without this history (*p* = 0.179). The 3- and 5-year competing risk-adjusted probabilities of grade ≥ 3 cardiac events in patients with preexisting ischemic heart disease were 25 and 27.5% compared with 7.2 and 7.2% in patients without this history (*p* = 0.014).

The median heart dose was 12 Gy. After adjustment for the competing risk of death, the 3- and 5-year rates of grade ≥ 2 events were 28.25 and 28.81% for patients with mean heart dose ≥ 12 Gy and 11.83 and 15.98% for patients with mean heart dose <12 Gy (*p* = 0.045; Figure 3A), and similar findings were noted for grade ≥ 3 cardiac events (*p* = 0.031, Figure 3B). For patients with preexisting ischemic heart disease, after adjustment for the competing risk of death, the 3- and 5-year probabilities of grade ≥ 2 cardiac events were 32.5 and 35% compared with 18.6 and 20.9%, respectively, in patients without this history (*p* = 0.179; Figure 4A), and there was a significant difference (*p* = 0.014) in the cumulative incidence of grade ≥ 3 cardiac events between patients with and without preexisting ischemic heart disease (Figure 4B).

### Survival

Median PFS and OS were 15 and 30 months, respectively. Two hundred sixty-four (76.3%) patients had disease progression. Five-year OS rate was 16.47%. On the multivariable analysis, disease progression (HR, 2.60; 95% CI, 1.82–3.70; *p* < 0.001), ECOG performance status (HR, 0.71; 95% CI, 0.56–0.91; *p* = 0.007), heart V5 (HR, 1.01; 95% CI, 1.00–1.03; *p* = 0.035), and GTV (HR, 1.00; 95% CI, 1.00–1.00; *p* = 0.020) were significant predictors of 5-year OS (Table 6). In the subgroup analysis, higher heart V5 (HR, 1.02; 95% CI, 1.00–1.03; *p* = 0.012) was significantly associated with worse OS for patients with lower tumor (*n* = 192), but for patients with upper tumor (*n* = 154), there was no statistically significant association between heart dosimetric parameters and OS (Table 7).

**Table 6:** Univariable and multivariable overall survival analyses.

**Characteristic**	**Univariable analysis**		**Multivariable analysis**	
	**HR (95% CI)**	***p*-value**	**HR (95% CI)**	***p*-value**
Male	1.14 (0.88–1.47)	0.329		
Age ≥ 66, year	1.02 (0.81–1.29)	0.857		
Current and former smoker	1.24 (0.99–1.56)	0.066	1.26 (0.99–1.60)	0.061
Alcoholic	1.21 (0.96–1.52)	0.115		
ECOG performance status (0 vs. ≥1)	0.84 (0.75–0.94)	0.003	0.71 (0.56–0.91)	0.007
Histology (squamous cell carcinoma vs. others)	0.80 (0.57–1.11)	0.185		
Chemotherapy	0.67 (0.49–0.91)	0.011	0.72 (0.52–0.99)	0.044
Tumor location (upper tumor vs. lower tumor)	1.26 (1.00–1.59)	0.050	0.74 (0.51–1.07)	0.111
^*^Disease progression	1.75 (1.47–2.07)	<0.001	2.60 (1.82–3.70)	<0.001
Treatment modality (3D-CRT vs. IMRT)	1.09 (0.85–1.39)	0.505		
Prescribed radiation dose, Gy	0.98 (0.95–1.01)	0.180		
Lung V5, %	1.02 (1.01–1.02)	<0.001	0.99 (0.97–1.02)	0.607
Lung V20, %	1.03 (1.01–1.04)	0.008	0.96 (0.92–1.01)	0.139
Mean lung dose, Gy	1.08 (1.04–1.12)	<0.001	1.11 (0.93–1.32)	0.251
Heart V5, %	1.01 (1.01–1.01)	<0.001	1.01 (1.00–1.03)	0.035
Heart V30, %	1.01 (1.01–1.02)	<0.001	1.02 (1.00–1.04)	0.100
Heart V60, %	1.02 (1.00–1.04)	0.041	0.98 (0.94–1.02)	0.289
Mean heart dose, Gy	1.02 (1.01–1.03)	<0.001	0.97 (0.92–1.02)	0.198
GTV, cm^3^	1.00 (1.00–1.00)	<0.001	1.00 (1.00–1.00)	0.020

*ECOG, Eastern Cooperative Oncology Group; 3D-CRT, three-dimensional conformal radiation therapy; IMRT, intensity-modulated radiation therapy; V5, volume received ≥ 5 Gy; V20, volume received ≥ 20 Gy; V30, volume received ≥ 30 Gy; V60, volume received ≥ 60 Gy; GTV, gross tumor volume; HR, hazard ratio; CI confidence interval*.

* *Time-dependent covariate*.

**Table 7:** Multivariable overall survival analysis for patients with upper and lower tumor location.

**Characteristic**	**Five-year overall survival (upper tumor)** **(*****n*** **=** **154)**		**Five-year overall survival (lower tumor)** **(*****n*** **=** **192)**	
	**HR (95% CI)**	***p*-value**	**HR (95% CI)**	***p*-value**
Current and former smoker	1.60 (1.11–2.31)	0.011		
ECOG performance status (0 vs. ≥1)			0.64 (0.46–0.89)	0.008
Chemotherapy	0.55 (0.34–0.91)	0.020	0.71 (0.45–1.11)	0.131
^*^Disease progression	5.54 (2.87–10.69)	<0.001	1.71 (1.10–2.65)	0.017
Lung V5, %	1.00 (0.99–1.02)	0.678	1.00 (0.98–1.02)	0.970
Lung V20, %			0.99 (0.94–1.05)	0.760
Mean lung dose, Gy			1.02 (0.84–1.24)	0.809
Heart V5, %	0.97 (0.93–1.02)	0.187	1.02 (1.00–1.04)	0.027
Heart V30, %	1.03 (0.95–1.13)	0.456	1.01 (0.99–1.04)	0.335
Heart V60, %	0.78 (0.54–1.13)	0.186		
Mean heart dose, Gy	1.14 (0.92–1.42)	0.231	0.96 (0.89–1.03)	0.248
GTV, cm^3^	1.00 (1.00–1.00)	0.281	1.00 (1.00–1.00)	0.090

*ECOG, Eastern Cooperative Oncology Group; V5, volume received ≥ 5 Gy; V20, volume received ≥ 20 Gy; V30, volume received ≥ 30 Gy; V60, volume received ≥ 60 Gy; GTV, gross tumor volume; HR, hazard ratio; CI, confidence interval*.

* *Time-dependent covariate*.

## Discussion

In this retrospective study of 346 patients with stage III esophageal cancer treated with definitive radiotherapy, we found that grade ≥ 2 cardiac events were often within the window of median expected survival for this patient population and demonstrated that grade ≥ 2 cardiac events were significantly associated with heart dose and occurred quite early after treatment. Although we set the dose limits and controlled the baseline cardiac risk, the heart dosimetric parameters were still significantly associated with cardiac events. Given the low 5-year OS rate, the actual incidence for cardiac toxicity is expected to be much higher. To the best of our knowledge, this is one of the largest detailed studies analyzing cardiac morbidity and assessing the relationship between heart dose and cardiac events or OS in patients with locally advanced esophageal cancer treated with definitive radiotherapy. These data provide meaningful insight for researchers and clinicians regarding clinical and dosimetric factors associated with increased hazard of developing clinically relevant cardiac complications.

Previous studies suggested the overall crude incidence of clinically relevant cardiac complications for esophageal cancer receiving radiotherapy was 10.8% (range: 5–44%), and 5.8–11.1% of the patients had grade 3 or higher cardiac toxicities that were considered clinically relevant (10). With a long median follow-up of 53 and 57 months, respectively, Ishikura et al. (15) and Kumekawa et al. (16) found that the incidence of grade ≥ 2 cardiac events was 11%. With a short median follow-up of 10.7 and 26.7 months, respectively, Morota et al. (17) and Konski et al. (18) found that 6 and 8% of patients developed grade ≥ 2 cardiac complications, respectively. Our study found that the rate of grade ≥ 2 cardiac events was higher that of than previous studies, probably because patients enrolled in our study were those who refused or could not tolerate surgery and because more than half of them (57.5%) had an ECOG score of 1 or above.

The standard definitive dose for locally advanced esophageal cancer is 50–50.4 Gy. However, in a recent study, patients with unresectable locally advanced esophageal cancer receiving chemoradiotherapy with a simultaneous integrated boost of radiotherapy dose (50.4 Gy to subclinical areas at risk and 63.0 Gy to the gross tumor and involved nodes) showed good tolerance and encouraging local control (19). In our study, patients also received a higher prescribed dose (median 60 Gy), which aimed to increase local control rates and OS. Previous studies have confirmed that compared with 3D-CRT, the use of IMRT is found to be significantly associated with lower all-cause mortality and cardiac mortality in patients with esophageal cancer (20). However, in this study, the incidence of cardiac events did not decrease in patients receiving IMRT, possibly owing to the differences in the years of patients receiving radiotherapy and the levels of radiotherapy techniques between these studies. Furthermore, although most patients in this study were treated with an advanced radiation delivery technique, IMRT, the incidence of cardiac implication was still high. These data may inform radiation oncologists that it is important to minimize heart dose even if advanced techniques are applied and more sophisticated techniques, such as proton therapy, are recommended to applied.

A common consensus is that cardiac events generally occurred 10 years or more after receiving treatment in patients with breast cancer or Hodgkin lymphoma (1–4). But Wang et al. (5) analyzed cardiac toxicity after radiotherapy for stage III non-small-cell lung cancer and reported that the first cardiac events that occurred within the first 3 years after treatment were found in 18 out of 26 patients (69.2%) with grade ≥ 2 cardiac events. Beukema et al. (10) summarized six researches reporting the incidence of radiation-induced cardiac toxicities for esophageal cancer and found that most events occurred within 2 years after treatment. This study found that of the 78 patients with grade ≥ 2 cardiac events, 70 (89.7%) had the first cardiac events that occurred within the first 3 years after treatment, which was consistent with previous studies.

Studies regarding whether dosimetric parameters could independently predict radiation-associated cardiac toxicities for patients with esophageal cancer are very limited. Ogino et al. (21) found that heart volume receiving ≥ 45 Gy (V45), heart volume receiving ≥ 50 Gy (V50), and heart volume receiving ≥ 55 Gy (V55) were independent risk factors of symptomatic cardiac disease in esophageal cancer patients receiving chemoradiotherapy. However, the median heart dose was only 27.5 Gy, significantly lower than the recommended radiation dose (50.4 Gy) in the National Comprehensive Cancer Network (NCCN) guidelines (22). This study showed that mean heart dose was significantly associated with a hazard of 1.12/Gy increase in the likelihood of developing a grade ≥ 2 cardiac event on the multivariable analysis. The subgroup analysis showed that the significant association between mean heart dose and grade ≥ 2 cardiac events existed only in patients with lower tumor, and none of dosimetric parameters were associated with grade ≥ 2 cardiac events for patients with upper tumor, because the overall heart doses for upper cases were very low and thus have a low number of cardiac events with which to detect a difference.

The general recommendation for tolerance dose limits of mean whole heart dose is ≤ 30 Gy for patients receiving thoracic radiotherapy (22, 23). However, in this report, of the 78 patients with grade ≥ 2 cardiac events, 58 (74.4%) had a mean heart dose of less than 30 Gy. These data provide the evidence that it is essential to minimize heart radiation exposure whenever possible to doses significantly lower than generally recommended in patients with lower esophageal cancer and to reduce the hazard of cardiac toxicity. However, in the current routine clinical practice, a reduction of the dose to the heart could usually generally result in a higher lung dose with an increased risk of radiation pneumonitis and fibrosis (10). Thus, future studies seeking the ideal balance between lung dose and heart dose are needed. Previous studies found that the most relevant dosimetric factors associated with cardiac events or OS for patients treated with thoracic radiation were diverse, such as mean heart dose, heart V45, heart V50, and heart V55, but did not appear to be the heart V5 (5, 6, 18, 21, 24, 25). Our study found that heart V5 was the most powerful factor to predict an increased hazard of grade ≥ 3 cardiac events and a worse OS, perhaps because the median heart V5 was fairly high (85.6%) in patients with lower esophageal cancer. Our study demonstrated that preexisting ischemic heart disease was significantly associated with a greater than 2-fold increase in the likelihood of developing a grade ≥ 2 cardiac event. For patients without preexisting ischemic heart disease, high WHO/ISH 10-year risk was significantly associated with a near 2-fold increase in the likelihood of developing of a grade ≥ 2 cardiac event. Therefore, patients with preexisting ischemic heart disease or with high WHO/ISH 10-year risk may need frequent monitoring of cardiac status with clinicians after radiotherapy.

The most frequently observed cardiac events were pericardial effusion, cardiac ischemia, and heart failure for esophageal cancer treated with thoracic radiation (10). In this study, the incidence of arrhythmia was high, ranking second in all cardiac events, perhaps owing to some arrhythmia events that occurred under an induction of other cardiac events. The interaction between cardiac events may lead to inaccuracies in estimating the incidence of cardiac toxicities authentically induced by radiotherapy. The rate of heart failure was relatively low, possibly owing to the comparative short-term follow-up.

There are several other limitations in this study. First, the retrospective nature may lead to a bias during patients' selection and an underestimation of the true cardiac events rates owing to the unrecorded cardiac toxicities. Second, the follow-up time was not long enough. This study assessed the incidence of cardiac events within 5 years after treatment, but after 5 or even 10 years, cardiac events may still occur. Third, the most relevant dose parameters for cardiac toxicities may be different. In this study, we only assessed the relationship between dosimetric parameters of whole heart and cardiac events, failing to acquire the dose–volume parameters of cardiac substructures including the left and right ventricles, pericardium, and coronary artery. Additionally, despite adopting the competing risk model, the high competing risk of cancer-related death was still a confounding factor for the incidence of cardiac events. Last, the chemotherapy regimens, prescribed doses, and fractionation schemes vary among patients in this study. Therefore, further prospective clinical trials with long follow-up period and uniform prescribed dose, fractionation, and chemotherapy are needed to confirm and broadly interpret the present findings.

In summary, heart dose was significantly associated with an increased cardiac event rate and a worse 5-year OS outcome for patients with stage III esophageal cancer treated with definitive radiotherapy. Most of first cardiac events occurred within the first 3 years after receiving treatment.

## Data Availability Statement

The datasets generated for this study are available on request to the corresponding author.

## Ethics Statement

The studies involving human participants were reviewed and approved by The Ethics Committee of Shandong Cancer Hospital Affiliated to Shandong First Medical University. The patients/participants provided their written informed consent to participate in this study. Written informed consent was obtained from the individual(s) for the publication of any potentially identifiable images or data included in this article.

## Author Contributions

XM and JY contributed to the conception and design of the study. GC and CL organized the database. GC performed the statistical analysis. GC, XM, and JY wrote the first draft of the manuscript. All authors contributed to the manuscript revision and read and approved the submitted version.

### Conflict of Interest

The authors declare that the research was conducted in the absence of any commercial or financial relationships that could be construed as a potential conflict of interest.
