# Supplementary material for: Heart Dosimetric Parameters Were Associated With Cardiac Events and Overall Survival for Patients With Locally Advanced Esophageal Cancer Receiving Definitive Radiotherapy
Source: Front Oncol. 2020 Mar 12;10:153. doi: 10.3389/fonc.2020.00153 (PMC7080859; doi:10.3389/fonc.2020.00153)
Supplement: Supplementary file 1 [file Table_1.doc]

[Supplementary Table 1](https://www.ncbi.nlm.nih.gov/pmc/articles/PMC6769083/#SM1) The details of patients with symptomatic cardiac events.

| Patients No. | Sex | Age (years) | Mean heart dose (Gy) | Overall survival (months) | Pre-existing ischemic heart disease | WHO/ISH 10-Year Risk (%) | Cardiac event details and time to cardiac event |
| --- | --- | --- | --- | --- | --- | --- | --- |
| 1 | Male | 70 | 15.35 | 2 | Yes | - | Grade 2: atrial fibrillation, 2 months |
| 2 | Male | 75 | 16.74 | 4 | No | 10-20 | Grade 2: atrial fibrillation, 3 months |
| 3 | Female | 71 | 31.36 | 38 | No | 20-30 | Grade 3: atrial fibrillation with Wolff -Parkinson-White syndrome, 3 months |
| 4 | Male | 75 | 31.89 | 15 | No | > 40 | Grade 3: unstable angina, 4 months |
| 5 | Male | 70 | 41.84 | 6 | No | 10-20 | Grade 2: pericardial effusion, 4 months |
| 6 | Female | 70 | 43.16 | 7 | Yes | - | Grade 2: pericardial effusion, 4 months |
| 7 | Male | 72 | 37.73 | 22 | No | 20-30 | Grade 3: heart failure, 5 months |
| 8 | Male | 71 | 21.91 | 5 | No | 20-30 | Grade 5: acute myocardial infarction, 5 months |
| 9 | Male | 72 | 30.23 | 7 | No | 30-40 | Grade 3: pericardial effusion, 5 months |
| 10 | Male | 73 | 20.72 | 51 | No | > 40 | Grade 3: sick sinus syndrome, 5 months |
| 11 | Male | 52 | 20.84 | 9 | No | 20-30 | Grade 2: pericardial effusion, 6 months |
| 12 | Male | 70 | 0.43 | 60 | Yes | - | Grade 2: pericardial effusion, 6 months; Grade 3: atrioventricular block, 6 months |
| 13 | Female | 69 | 40.98 | 7 | No | 30-40 | Grade 5: acute myocardial infarction, 7 months |
| 14 | Male | 56 | 16.92 | 10 | No | 20-30 | Grade 2: pericardial effusion, 7 months |
| 15 | Male | 72 | 3.26 | 10 | No | 10-20 | Grade 2: pericardial effusion, 7 months |
| 16 | Female | 76 | 29.83 | 11 | No | > 40 | Grade 2: pericardial effusion, 7 months |
| 17 | Male | 55 | 39.62 | 11 | No | 20-30 | Grade 2: pericardial effusion, 7 months |
| 18 | Male | 70 | 19.20 | 43 | No | 20-30 | Grade 3: acute pericarditis, 8 months |
| 19 | Male | 71 | 27.00 | 17 | No | > 40 | Grade 3: unstable angina, 8 months |
| 20 | Male | 73 | 37.80 | 25 | No | 30-40 | Grade 3: unstable angina, 8 months |
| 21 | Male | 72 | 35.53 | 19 | No | 20-30 | Grade 3: acute pericarditis, 8 months |
| 22 | Female | 66 | 15.90 | 21 | No | 20-30 | Grade 3: pericardial effusion, 8 months |
| 23 | Female | 75 | 33.11 | 26 | Yes | - | Grade 3: unstable angina, 8 months |
| 24 | Female | 71 | 33.21 | 18 | No | 30-40 | Grade 3: unstable angina, 8 months |
| 25 | Male | 70 | 2.36 | 11 | No | 20-30 | Grade 2: left bundle branch block, 8 months |
| 26 | Male | 62 | 48.20 | 11 | No | 20-30 | Grade 2: pericardial effusion, 9 months |
| 27 | Female | 76 | 46.62 | 12 | Yes | - | Grade 2: atrial fibrillation, 9 months |
| 28 | Male | 59 | 1.37 | 11 | No | < 10 | Grade 2: pericardial effusion, 10 months |
| 29 | Male | 76 | 19.78 | 23 | Yes | - | Grade 3: unstable angina, 10 months |
| 30 | Female | 74 | 21.47 | 12 | No | 20-30 | Grade 2: pericardial effusion, 11 months |
| 31 | Male | 65 | 18.72 | 35 | No | 20-30 | Grade 3: unstable angina, 11 months |
| 32 | Male | 56 | 21.55 | 14 | No | 10-20 | Grade 3: heart failure, 11 months |
| 33 | Male | 58 | 26.52 | 20 | No | 10-20 | Grade 2: pericardial effusion, 11 months |
| 34 | Female | 65 | 25.04 | 21 | Yes | - | Grade 2: atrial fibrillation, 11 months; Grade 3: acute myocardial infarction, 12 months; Grade 3: ventricular tachycardia, 12 months; Grade 5: heart failure, 21 months |
| 35 | Female | 67 | 20.10 | 12 | No | 20-30 | Grade 2: pericardial effusion, 12 months |
| 36 | Male | 73 | 0.77 | 60 | No | 10-20 | Grade 2: pericardial effusion, 12 months |
| 37 | Male | 58 | 31.72 | 19 | Yes | - | Grade 3: heart failure, 13 months |
| 38 | Female | 70 | 4.39 | 60 | No | 10-20 | Grade 2: pericardial effusion, 13 months |
| 39 | Male | 57 | 25.46 | 16 | No | 20-30 | Grade 2: Wolff -Parkinson-White syndrome, 14 months |
| 40 | Male | 50 | 17.50 | 18 | No | 20-30 | Grade 2: atrial flutter, 14 months |
| 41 | Male | 59 | 22.19 | 40 | Yes | - | Grade 3: atrial fibrillation, 14 months |
| 42 | Female | 77 | 30.38 | 60 | No | 10-20 | Grade 2: atrioventricular block, 14 months |
| 43 | Male | 54 | 31.93 | 20 | No | 10-20 | Grade 3: acute myocardial infarction, 14 months; Grade 4: ventricular fibrillation, 16 months |
| 44 | Male | 51 | 1.66 | 21 | No | 10-20 | Grade 2: pericardial effusion, 15 months |
| 45 | Male | 77 | 8.00 | 39 | No | 30-40 | Grade 2: pericardial effusion, 15 months |
| 46 | Male | 65 | 27.24 | 32 | Yes | - | Grade 3: unstable angina, 15 months; Grade 2: pericardial effusion, 25 months; Grade 5: heart failure, 30 months |
| 47 | Male | 65 | 32.88 | 19 | No | 10-20 | Grade 2: pericardial effusion, 16 months |
| 48 | Male | 67 | 18.25 | 20 | No | 20-30 | Grade 2: pericardial effusion, 16 months |
| 49 | Male | 64 | 31.73 | 23 | No | > 40 | Grade 4: acute myocardial infarction, 16 months |
| 50 | Male | 54 | 34.13 | 26 | No | 20-30 | Grade 3: aortic insufficiency, 16 months |
| 51 | Male | 60 | 3.55 | 23 | No | 10-20 | Grade 2: paroxysmal supraventricular tachycardia, 16 months |
| 52 | Female | 79 | 0.70 | 42 | No | 20-30 | Grade 2: pericardial effusion, 16 months |
| 53 | Male | 44 | 6.12 | 49 | No | 20-30 | Grade 2: aortic stenosis, 16 months |
| 54 | Male | 50 | 29.43 | 18 | No | 10-20 | Grade 3: acute myocardial infarction, 17 months; Grade 5: ventricular fibrillation, 18 months |
| 55 | Male | 79 | 3.52 | 40 | No | 30-40 | Grade 2: atrial fibrillation, 18 months |
| 56 | Male | 61 | 34.72 | 39 | Yes | - | Grade 2: pericardial effusion, 18 months; Grade 3: unstable angina, 33 months; Grade 3: ventricular tachycardia, 34 months |
| 57 | Male | 69 | 2.31 | 49 | No | 10-20 | Grade 2: aortic insufficiency, 19 months |
| 58 | Female | 60 | 0.96 | 47 | No | 20-30 | Grade 2: heart failure, 20 months |
| 59 | Male | 57 | 12.08 | 48 | No | 10-20 | Grade 2: mitral stenosis, 21 months |
| 60 | Female | 43 | 20.27 | 28 | No | < 10 | Grade 2: aortic stenosis combined with insufficiency, 22 months |
| 61 | Male | 49 | 1.10 | 39 | No | 20-30 | Grade 2: heart failure, 22 months |
| 62 | Male | 69 | 3.86 | 40 | No | 30-40 | Grade 2: aortic stenosis, 22 months |
| 63 | Male | 66 | 0.41 | 60 | Yes | - | Grade 3: unstable angina, 25 months; Grade 3: acute myocardial infarction, 40 months |
| 64 | Female | 68 | 3.96 | 39 | No | 10-20 | Grade 2: pericarditis, 26 months |
| 65 | Female | 63 | 27.16 | 39 | No | 20-30 | Grade 3: myocarditis, 29 months |
| 66 | Female | 81 | 5.26 | 38 | No | 10-20 | Grade 2: aortic insufficiency, 30 months |
| 67 | Male | 56 | 15.87 | 37 | No | 20-30 | Grade 3: aortic stenosis combined with insufficiency, 30 months; Grade 2:left bundle branch block, 30 months |
| 68 | Female | 63 | 25.40 | 41 | Yes | - | Grade 3: unstable angina, 30 months; Grade 2: right bundle branch block, 33 months |
| 69 | Male | 60 | 4.17 | 40 | No | 20-30 | Grade 2: atrial fibrillation, 33 months |
| 70 | Male | 74 | 21.44 | 38 | No | > 40 | Grade 4: pericardial tamponade, 34 months |
| 71 | Male | 69 | 19.21 | 44 | No | 20-30 | Grade 2: mitral insufficiency, 38 months |
| 72 | Male | 73 | 6.95 | 42 | No | 20-30 | Grade 2: unstable angina, 39 months |
| 73 | Male | 50 | 0.79 | 46 | No | 20-30 | Grade 2: mitral insufficiency and aortic insufficiency, 40 months |
| 74 | Female | 70 | 9.51 | 60 | No | 10-20 | Grade 2: atrial fibrillation, 45 months |
| 75 | Male | 57 | 0.98 | 60 | Yes | - | Grade 3: mitral stenosis and aortic insufficiency, 51 months |
| 76 | Female | 69 | 3.12 | 60 | No | < 10 | Grade 2: pericardial effusion, 52 months |
| 77 | Male | 69 | 1.26 | 60 | No | 10-20 | Grade 2: heart failure, 57 months |
| 78 | Male | 65 | 0.57 | 60 | No | 20-30 | Grade 2: aortic stenosis, 58 months |

Abbreviations: WHO/ISH, World Health Organization/International Society of Hypertension.
